# Supplementary figures and images for: Transcriptomic profiles from normal and tumor tissue samples reveal distinct venule populations and novel tumor endothelial cell markers in breast cancer
Source: Breast Cancer Res. 2026 Mar 3;28:69. doi: 10.1186/s13058-026-02249-0 (PMC13063865; doi:10.1186/s13058-026-02249-0)

# Supplemental Figure 1

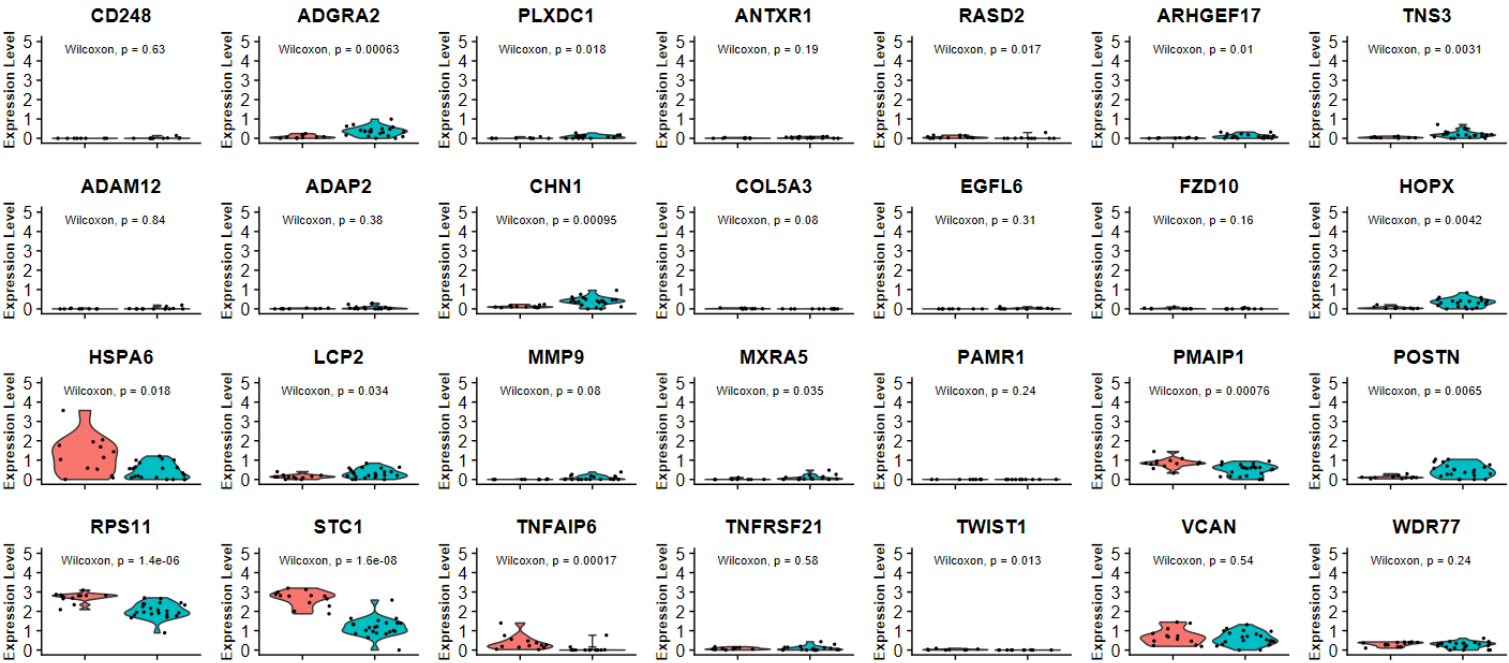

Supplement: Supplementary file 3 — Additional file3 (PDF 542 KB) Supplemental Figure 1 Expression of the previously identified lung cancer and colon cancer TEMs in breast NEC and TEC. P-values were determined by the Wilcoxon rank sum test. [file 13058_2026_2249_MOESM3_ESM.pdf]

# Supplemental Figure 2

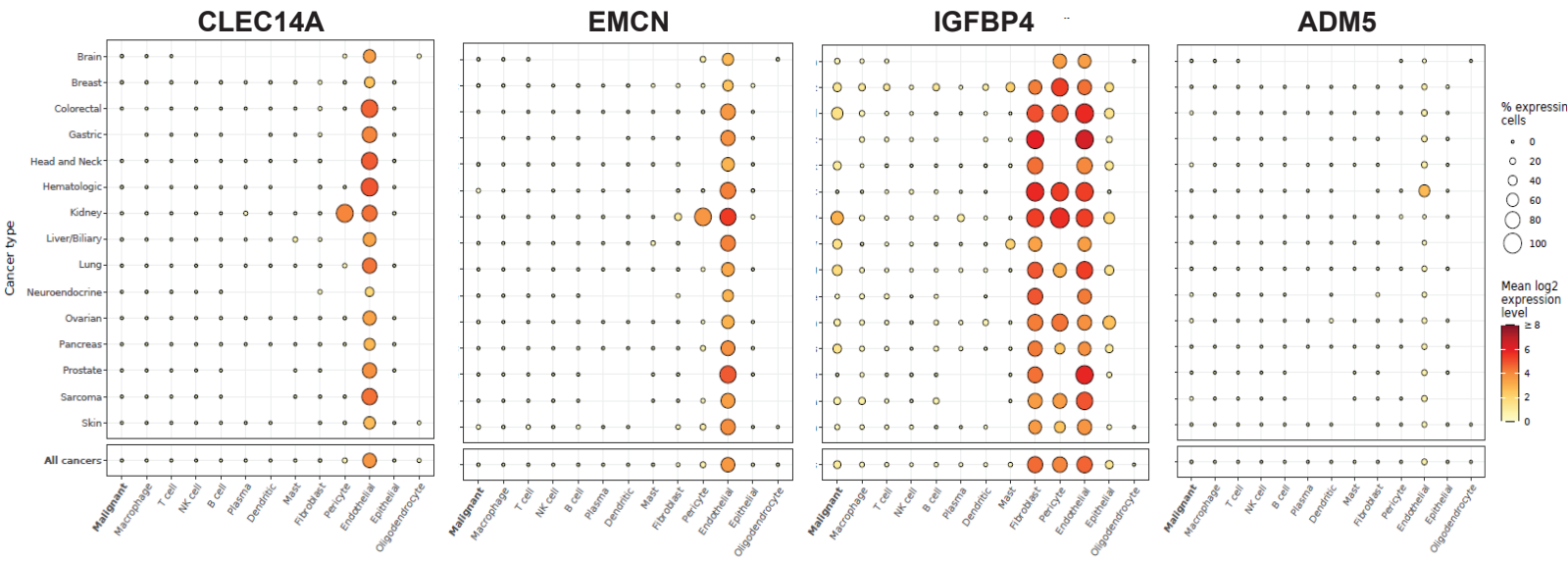

Supplement: Supplementary file 4 — Additional file4 (PDF 743 KB) Supplemental Figure 2 Cell type-specific expression of CLEC14A, EMCN, IGFBP4, and ADM5 in the 3CA pan-cancer single-cell dataset. Dot color reﬂects gene expression level and dot size represents the percentage of cells expressing genes within each cluster. [file 13058_2026_2249_MOESM4_ESM.pdf]

# Supplemental Figure 3

## Breast Cancer - all

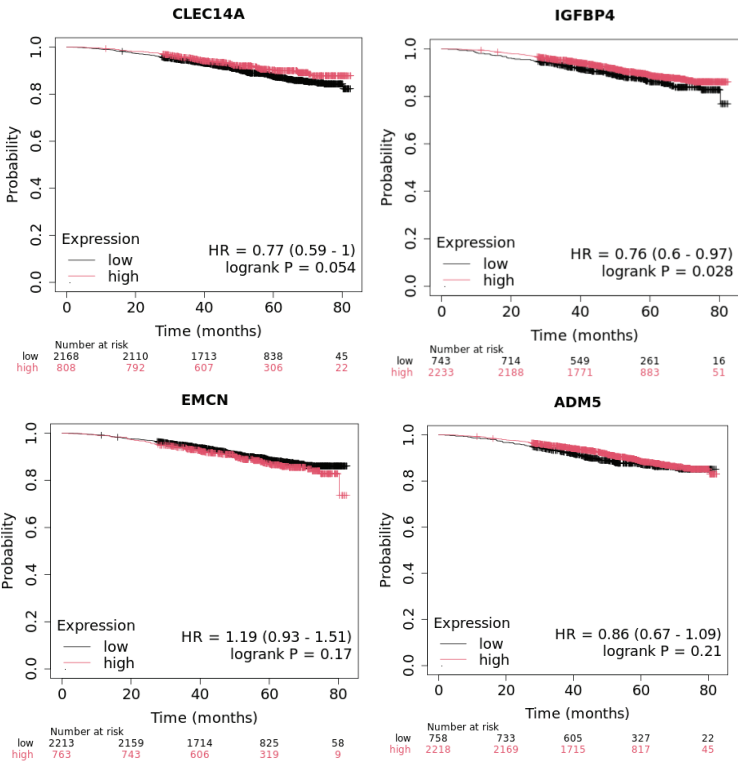

Supplement: Supplementary file 5 — Additional file5 (PDF 703 KB) Supplemental Figure 3 Correlation of CLEC14A, EMCN, IGFBP4, and ADM5 gene expression to overall survival in breast cancer patients (n=2976). Cox proportional hazards regression analysis was used to calculate hazard ratio (HR) h with 95% confidence intervals and log-rank p-value. [file 13058_2026_2249_MOESM5_ESM.pdf]

## Supplemental Figure 4

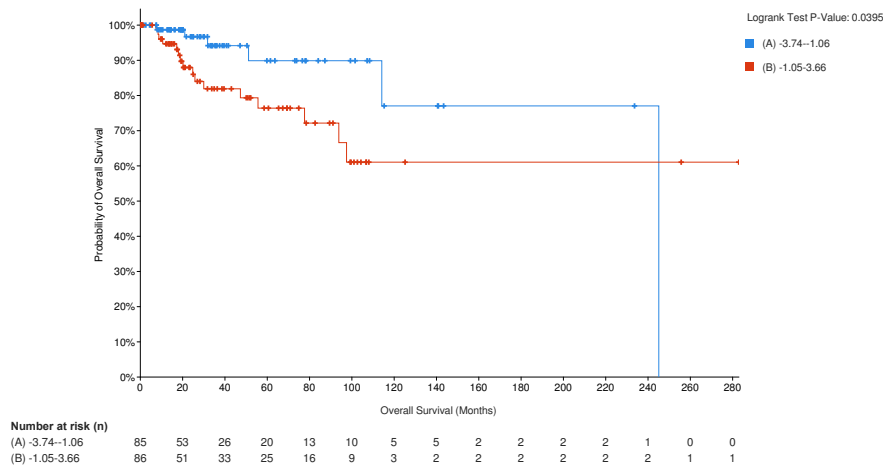

Supplement: Supplementary file 6 — Additional file6 (PDF 439 KB) Supplemental Figure 4 Correlation of ADM5 gene expression to overall survival in breast cancer patients with the PAM50 basal subtype (n=171) from the TCGA – BRCA dataset. Cox proportional hazards regression analysis was used to calculate hazard ratio (HR) h with 95% confidence intervals and log-rank p-value. [file 13058_2026_2249_MOESM6_ESM.pdf]

# Supplemental Figure 5

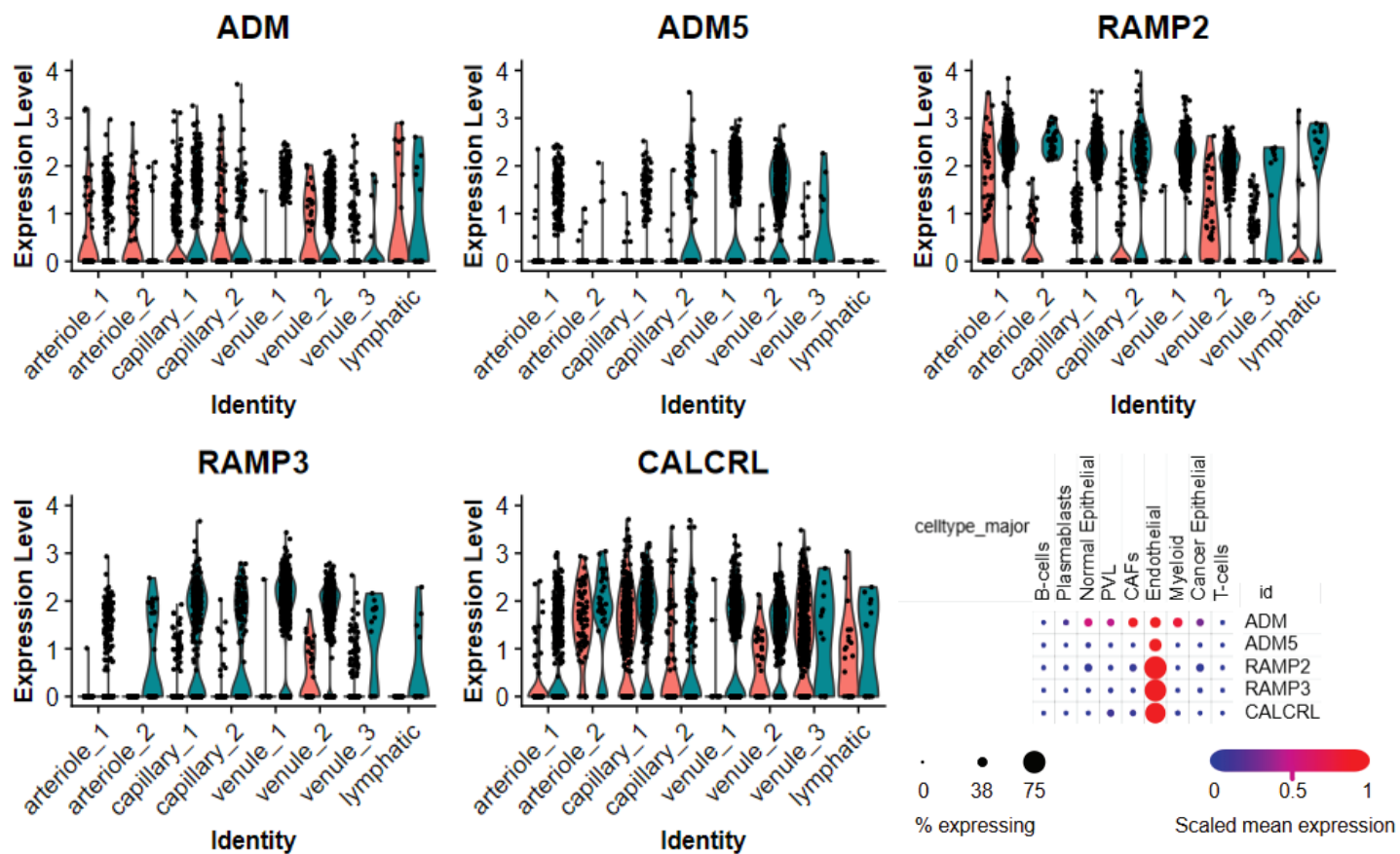

Supplement: Supplementary file 7 — Additional file7 (PDF 610 KB) Supplemental Figure 5 Expression of the calcitonin gene-related peptide family and related receptors. Expression of ADM, ADM5, RAMP2, RAMP3, and CALCRL. EC cluster expression split by tissue source (NEC and TEC). Cell type-specific expression of ADM, CARCRL, RAMP2, and RAMP3 in the initial Wu et al major cell type clusters. Dot color reﬂects gene expression level and dot size represents the percentage of cells expressing genes within each cluster. [file 13058_2026_2249_MOESM7_ESM.pdf]
